# Penguins are competent hosts of *Haemoproteus* parasites: the first detection of gametocytes, with molecular characterization of *Haemoproteus larae*

**DOI:** 10.1186/s13071-020-04176-1

**Published:** 2020-06-12

**Authors:** Mizue Inumaru, Shiori Aratani, Misa Shimizu, Mineka Yamamoto, Yukita Sato, Koichi Murata, Gediminas Valkiūnas

**Affiliations:** 1 Laboratory of Biomedical Science, Department of Veterinary Medicine, College of Bioresource Sciences, Nihon University, Fujisawa, 252-0880 Japan; 2 Laboratory of Wildlife Science, Department of Animal Science and Resources, College of Bioresource Sciences, Nihon University, Fujisawa, 252-0880 Japan; 3 Nature Research Centre, Akademijos 2, 08412 Vilnius, Lithuania

**Keywords:** *Haemoproteus*, Cytochrome *b* lineage, Molecular characterization, Penguin, gametocyte, Japan

## Abstract

**Background:**

The majority of penguins (Sphenisciformes) have evolved in areas with weak or absent transmission of haemosporidian parasites and are usually naïve to avian haemosporidian infections. *Plasmodium* parasites are transmitted by mosquitoes, and lethal avian malaria has been often reported in captive penguins in many countries. The related haemosporidian parasites belonging to *Haemoproteus* and *Leucocytozoon* have also been detected in penguins but less often than *Plasmodium* infections. The majority of *Haemoproteus* infection reports in penguins are based solely on PCR-based diagnostics. It remains unclear if haemoproteids can complete their life-cycle and produce infective stages (gametocytes) in penguins or whether these infections are abortive in penguins, and thus dead ends for transmission. In other words, it remains unknown if penguins are competent hosts for *Haemoproteus* parasites, which cause disease in non-adapted birds.

**Methods:**

Two captive African penguins (*Spheniscus demersus*) and two Magellanic penguins (*S. magellanicus*) were found to be positive for *Haemoproteus* infection in two open-air aquariums in Japan, and the parasites were investigated using both PCR-based testing and microscopical examination of blood films. Samples from a black-tailed gull (*Larus crassirostris*) and previously tested gulls were used for comparison.

**Results:**

The lineage hSPMAG12 was detected, and gametocytes of *Haemoproteus* sp. were seen in the examined penguins and gull. Observed gametocytes were indistinguishable from those of *Haemoproteus larae*, which naturally parasitize birds of the genus *Larus* (Laridae). The detected sequence information and Bayesian phylogenetic analysis supported this conclusion. Additionally, morphologically similar gametocytes and closely related DNA sequences were also found in other gull species in Japan. Phylogenetic analysis based on partial *cytb* sequences placed the lineage hSPMAG12 of *H. larae* within the clade of avian haemoproteids which belong to the subgenus *Parahaemoproteus*, indicating that *Culicoides* biting midges likely transmit the parasites between penguins and gulls.

**Conclusions:**

This study shows that some species of *Haemoproteus* parasites complete their development and produce gametocytes in penguins, which may be source of infection for biting midges transmitting haemoproteosis. To prevent haemosporidiosis in zoos, we call for control not only of mosquitoes, but also biting midges.
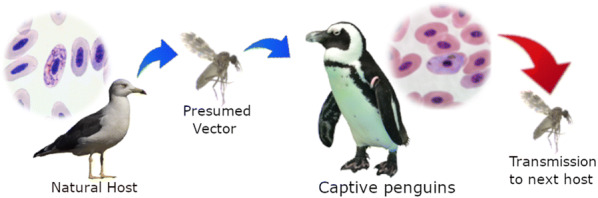

## Background

Penguins (Sphenisciformes) are flightless bird species well known for their unique upright posture and outstanding swimming abilities. Many species are recognized to be near extinction [1]. To conserve these non-flying birds, their present status of risk for extinction should be considered from various aspects, including infectious diseases [2, 3]. The majority of penguins inhabit areas free of haemosporidian parasites or of low prevalence and thus are usually naïve to these infections. The first case of avian haemosporidian infection was the *Plasmodium* parasite reported in a captive king penguin (*Aptenodytes patagonicus*) at London Zoo in 1926 [4]. Since then, lethal cases of *Plasmodium* infections have often been reported in captive penguins in many countries [5, 6]. These reports came from captive penguins which were usually maintained in open-air aviaries in zoos or aquariums where free access of mosquito vectors was possible. *Plasmodium* parasites cause malaria, which is transmitted by hematophagous mosquitoes carrying these pathogens from infected wild birds living around the penguin enclosures [7, 8]. Japan has one of the world’s largest *ex-situ* populations of penguins, with over 3000 penguin individuals at zoos and aquariums, including nearly 500 African penguins (*Spheniscus demersus*) [9, 10]. Avian malaria has been sporadically detected in penguins in Japan [11]. Furthermore, blood of *S. demersus* has been detected in *Culex pipiens pallens*, which is one of the major vectors of avian malaria in Japan [12].

The related haemosporidian parasites belonging to the genera *Haemoproteus* and *Leucocytozoon* have also been detected in penguins, but less often compared to avian *Plasmodium* [6]. *Leucocytozoon tawaki* has been described from Fiordland penguins (*Eudyptes pachyrhynchus*) and a New Zealand little penguin (*Eudyptula minor*), as well as from its vectors, blackflies (Simuliidae) [13, 14]. *Leucocytozoon* spp. parasites have also been detected in African penguins, yellow-eyed penguins (*Megadyptes antipodes*), and macaroni penguins (*Eudyptes chrysolophus*) [6, 15–17]. Death of penguins, particularly chicks and juveniles, due to *Leucocytozoon* infection suggested that leucocytozoonosis might be highly virulent in penguins [15]. Meanwhile, gametocytes of *Leucocytozoon* parasites have been detected from Fiordland and African penguins [13, 16], suggesting that the parasites are able to complete the life-cycle in these birds, making the transmission of leucocytozoonosis between penguins *via* vectors possible [8].

The majority of *Haemoproteus* infection reports in penguins are based solely on PCR-based diagnostics [6, 18, 19], with only two morphological observations of tissue stages suspected to correspond to *Haemoproteus* sp. [20, 21]. Meanwhile, there have been no reports of *Haemoproteus* spp. gametocytes in penguins [6]. Contrary to *Plasmodium* and *Leucocytozoon* parasites, it remains unclear if haemoproteids complete development and produce infective stages (gametocytes) or whether these infections are abortive at sporozoite or tissue stages in penguins, and the resulting infections are dead ends for parasite transmission. In other words, it remains unclear if penguins are competent hosts for *Haemoproteus* parasites, which are known to cause severe disease in non-adapted avian hosts [22, 23]. Unless gametocytes are present in the bloodstream, arthropod vectors are not capable of transmitting the parasite to another host. *Haemoproteus* species have generally been considered relatively harmless in the past [24], but cases of lethal haemoproteosis have been reported in captive birds [22, 23, 25, 26] and haemoproteosis may possibly be lethal to penguins as well.

In order to consider pathogenicity of haemoproteosis, a better understanding of penguins’ competence to *Haemoproteus* species is necessary. This knowledge would help improve conservation of these unique and endangered birds. To the best of our knowledge, this study reports the first case of *Haemoproteus* sp. gametocytes in penguins, suggesting that these birds are not only susceptible to haemoproteosis but are also competent hosts for some *Haemoproteus* species and may possibly be reservoirs of these pathogens. This paper provides molecular and morphological characterization of haemoproteids parasitizing penguins in Japan.

## Methods

### Samples

Two African penguins kept in aquarium A and two Magellanic penguins (*S. magellanicus*) in aquarium B were found to be infected with haemosporidians in 2017 and 2018, respectively (Table 1). Both aquariums are located in central Japan, and the penguins were maintained outdoors for exhibition. The birds were in good health during this study. All penguins had been inoculated with antimalarial prophylactic medications prior to and during sampling, but detailed protocols of the treatment were not provided by the facilities. Blood samples were obtained from the metatarsal vein, placed in microtubes containing 70% ethanol and sent to Nihon University College of Bioresource Sciences Laboratory of Biomedical Science, Fujisawa, Japan, where they were kept at -20 °C until further processing. Directly after withdrawal of blood, the blood samples were also used to prepare two blood films per individual, which were rapidly air-dried and sent to the laboratory, where they were fixed with methanol and stained with Hemacolor® (Merck KGaA, Darmstadt, Germany). Upon confirmation that the preparations were dry after staining, the blood films were mounted in Eukitt medium (O. Kindler GmbH, Freiburg, Germany).

**Table 1:** Information about samples used in this study and results of material examination

Facility	Bird individual number	Bird species	Sample No.	Date of hatching	Sampling date	PCR testing^a^		Microscopic examination	
						50 ng	200 ng	Parasitemia	Intensity of parasitemia
Aquarium A	1	*Spheniscus demersus*^c^	1a	2007-02-08	2016-08-04	–^b^	+	–	
			1b		2017-07-03	+	+	+	< 1/10,000
			1c		2017-09-15	–	+	–	
			1d		2017-11-06	–	+	–	
	2	*Spheniscus demersus*	2a	2008-11-14	2017-04-29	+	+	+	< 1/10,000
			2b		2017-11-17	–	+	+	< 1/10,000
Aquarium B	3	*Spheniscus magellanicus*	3	2009-04-25	2018-08-15	+	+	+	< 1/10,000
	4	*Spheniscus magellanicus*	4	2012-05-20	2018-08-13	+	+	+	< 1/10,000
Gyotoku Wild	5	*Larus crassirostris*	5a	2016-09-22^d^	2016-10-09	+	nd	+	10/1000
Bird Hospital			5b		2017-01-08	+	nd	+	12/1000

^a^50 ng and 200 ng refer to the amount of DNA template included in the PCR reactions

^b^Results: +, positive; -, negative; nd, no data (untested)

^c^Samples for individuals no. 1, no. 2 and no. 5 are given in order of sampling date

^d^Date of rescue and admittance to the facility. Date of hatching is unknown

In addition, one black-tailed gull (*Larus crassirostris*) was examined as part of a different on-going study (see [27]) (Table 1). The rescued bird was taken to Gyotoku Wild Bird Hospital, Chiba, Japan, where it was sampled twice (in October 2016 and January 2017). All blood samples were obtained and processed for examination using the same method as the penguin samples. These samples were used for comparison with parasites found in penguins.

### Molecular analysis

DNA was extracted from the blood samples using a standard phenol-chloroform method and dissolved in Tris-EDTA buffer. DNA concentration and quality were determined using a Nanodrop One Microvolume UV-Vis Spectrophotometer (Thermo Fisher Scientific, MA, USA). DNA concentration was then adjusted to 50 ng/µl for the black-tailed gull samples and to two different concentrations, 50 ng/µl and 200 ng/µl, for the penguin samples.

Polymerase chain reaction (PCR) targeting the partial mitochondrial cytochrome *b* (*cytb*) gene of avian haemosporidian parasites was run according to a previously described protocol [28]. Briefly, the first PCR was carried out using the HaemNFI/HaemNR3 primer set. Then, HaemF/HaemR2 and HaemFL/HaemR2L primer sets were used to amplify *Plasmodium* spp./*Haemoproteus* spp. and *Leucocytozoon* spp. DNA, respectively. The final reaction volume was 25 µl each, containing 2 mM MgCl_2_, 0.2 mM deoxynucleotide triphosphate, 10× ExTaq Mg^2+^ free buffer (TaKaRa, Ohtsu, Japan), 0.625 U Ex-Taq (TaKaRa), 10 µM of each primer and DNA template. In order to test the sensitivity and to identify any false-negatives, the PCR for the penguin samples was tested with both 50 ng and 200 ng of DNA template. PCR conditions were followed according to Hellgren et al. [28]. For the black-tailed gull samples, PCR was only tested with 50 ng of DNA template. As a positive control, 50 ng of DNA template of *Plasmodium gallinaceum* pGALLUS01 derived from an experimentally infected chicken (*Gallus gallus*) was used. Negative controls were also included using distilled water instead of DNA.

To confirm amplification, 1.5% agarose gels (Agarose S; Nippon Gene, Tokyo, Japan) containing ethidium bromide (Nacalai Tesque, Kyoto, Japan) were placed in chambers filled with TAE buffer, and PCR products were loaded into the wells with one positive control and one negative control per gel. Electrophoresis was performed at 100 V for approximately 20 min, gels were then placed under ultraviolet light to visualize the bands. Positive bands were cut from the gel and DNA was extracted using thermostable β-agarase (Nippon Gene, Tokyo, Japan).

The extracted DNA was directly sequenced in both directions with BigDye^TM^ terminator cycle sequence kit 3.1 (Applied Biosystems, Forster City, CA, USA) and ABI 3130-Avant Auto Sequencer (Applied Biosystems). After contig assembly, the obtained 478 bp nucleotide sequences were compared with sequences in the GenBank database using BLAST [29] and in the MalAvi database [30].

A Bayesian phylogeny was constructed using Mr Bayes version 3.2 [31]. The following sequences were included in the alignment: (i) lineages of morphologically characterised *Haemoproteus* and *Plasmodium* species; (ii) *Haemoproteus* spp. lineages previously detected from penguins (Table 2); (iii) *Haemoproteus* spp. lineages previously detected from gulls (*Larus* spp.) (Table 2); and (iv) closely related *Haemoproteus* spp. lineages to the lineage detected in this study based on pairwise distance. Morphologically characterised haemosporidian species were selected in reference to Chagas et al. [32]. *Leucocytozoon* sp. lSISKIN2 was used as the outgroup. Genetic distance between lineages was calculated by implementing the Jukes-Cantor model of substitution in MEGA7 [33]. Comparisons between lineages was also made using translated amino acid sequences as implemented in MEGA7 [33].

**Table 2:** Lineages of *Haemoproteus* previously detected from penguins and *Larus* gulls

	Lineage^a^	GenBank ID	Host species	Status	Continent	Country	Locality	References
*Haemoproteus* lineages from penguins (Sphenisciformes)	APPAT01	AB604312	*Aptenodytes patagonicus*	Captive	Asia	Japan	Hokkaido	Sasaki et al. (unpublished)
	DIGCAE01^c^	KC121057	*Spheniscus mendiculus*	Wild	South America	Ecuador	Galapagos	[21]
	EUMIN01^c^	KC121056	*Eudyptula minor*	Wild	Australia	Australia	Penguin Island	[21]
	EUMIN02^c^	KC121053	*Eudyptula minor*	Wild	Australia	Australia	Penguin Island	[21]
	HYPHI07	AB604313	*Eudyptes chrysocome*	Captive	Asia	Japan	Hokkaido	Sasaki et al. (unpublished)
	PE101^b,d^	KJ561806	*Spheniscus humboldti*	Wild	South America	Peru	Punta San Juan	[19]
	PE112^b,d^	KJ561807	*Spheniscus humboldti*	Wild	South America	Peru	Punta San Juan	[19]
	SPHUM04	AB604311	*Spheniscus humboldti*	Captive	Asia	Japan	Kanagawa	Sasaki et al. (unpublished)
	SPMAG12	AB604310	*Spheniscus magellanicus*	Captive	Asia	Japan	Miyagi	Sasaki et al. (unpublished)
			*Spheniscus demersus*	Captive	Asia	Japan		Present study
			*Spheniscus magellanicus*	Captive	Asia	Japan		Present study
	SPMEN02^d^	GQ395686	*Spheniscus mendiculus*	Wild	South America	Ecuador	Galapagos	[18]
*Haemoproteus* linages from *Larus* spp.	Anja3D^b,d^	GQ404558	*Larus scoresbii*	Wild	Europe	UK	New Island	[67]
	LARCAC01^d^	AF465593	*Larus cachinnans*	Wild	Europe	Spain		[68]
	LARCRA01	EF380176	*Larus crassirostris*	Wild	Asia	South Korea		[69]
			*Larus crassirostris*	Captive	Asia	Japan	Chiba	[27]
			*Larus canus*	Captive	Asia	Japan	Chiba	[27]
	LARCRA02	LC230123	*Larus crassirostris*	Captive	Asia	Japan	Chiba	[27]

^a^Codes of lineages are given according to MalAvi database, unless otherwise denoted

^b^Codes of lineages are given according to the GenBank database metadata

^c^There is debate on the validity of these records, see text

^d^Lineages shorter than 400 bp in the partial *cytb* gene sequence investigated in this study; these were excluded from phylogenetic analysis

Using jModelTest2 [34], the General Time Reversible model with gamma distribution for variable sites and proportion of sites as invariable (GTR+Γ+I) was selected as the best-fit model under the Bayesian information criterion. Markov chain Monte Carlo (MCMC) sampling was run independently twice for three million generations, with a sampling frequency of every 1000 generations [31]. The first 25% trees were discarded as “burn-in”. The resulting phylogenetic tree was visualized using FigTree 1.4 [35].

The entire process of screening for avian haemosporidians from DNA extraction to genetic analysis was repeated twice, each time by a different person in order to confirm for any possible contaminations or experimental errors. Furthermore, the samples from penguins and from the black-tailed gull were individually processed separately and in different months. Thus, a possibility of contamination between these samples was ruled out.

### Microscopical examination

Blood films were examined using an Olympus BX43 light microscope (Olympus, Tokyo, Japan). Two blood films were examined carefully for each blood sample. Entire blood films from each penguin were screened at 400× magnification and then at 1000× magnification. Each penguin blood film was examined for approximately 10 h. Blood films from the black-tailed gull were screened at the same magnifications. If there were any suspicious findings, the smear was observed using an Olympus IX71 light microscope (Olympus) and photographed with cellSens Standard 1.6 (Olympus). The detected haemosporidians were identified based on morphological features of their blood stages [8].

Intensity of parasitemia was calculated in all blood films at 400× magnification by actual counting of the number of parasites per 1000 erythrocytes or 10,000 erythrocytes in case of low parasitemia, starting at a random location.

Gametocytes of penguin *Haemoproteus* parasites were compared to those of the closely related lineages found in birds sampled at a wild bird rescue facility in East Japan [27]. In addition to gametocytes of the black-tailed gull sampled during this study (see above), gametocytes of the following *Haemoproteus* lineages were re-examined and used for comparison with penguin parasites: the lineage hNUMPHA01 from one whimbrel (*Numenius phaeopus*); the lineage hLARCRA01 from one mew gull (*Larus canus*) and one black-tailed gull; and the lineage hLARCRA02 from one black-tailed gull. All of these samples were collected during our previous study [27]. Furthermore, voucher blood films (accessions 1526.86-1527.86, Nature Research Centre, Vilnius) with gametocytes of *Haemoproteus larae* in the type avian host black-headed gull (*Larus ridibundus*) sampled at the type-locality (southern Kazakhstan) were examined and compared with parasites reported in penguins, gulls and whimbrel during this study.

Representative blood films with gametocytes from penguins (MPM Coll. No. 21620-21624), mew gull (MPM Coll. No. 21625), black-tailed gull (MPM Coll. No. 21626-21629) and whimbrel (MPM Coll. No. 21630) were deposited in the Meguro Parasitological Museum, Tokyo, Japan.

### Molecular test for possible co-infections

In order to rule out possible co-infections of haemosporidians belonging to *Plasmodium* and *Haemoproteus*, a nested-multiplex PCR was run for all parasite-positive penguin samples using 200 ng DNA template as described previously [36, 37]. First, a primary PCR was carried out using primers AE298/AE299 targeting a portion of mitochondrial *cox*1 gene and the full *cytb* gene [36]. The PCR reaction contained 4 mM MgCl_2_, 0.2 mM deoxynucleotide triphosphate, 10× ExTaq Mg^2+^ free buffer (Takara, Ohtsu, Japan), 1.0 U Ex-Taq (Takara), 10 µM of each primer and 200 ng DNA template, with a final reaction volume of 25 µl each. The PCR amplification was carried out according to Pacheco et al. [36]. The second PCR was carried out according to a previously described protocol using primers PMF/PMR and HMF/HMR, targeting an inner region of *cytb* [37]. This multiplex PCR reaction contained 5 µl of commercial master mix (2× Qiagen Multiplex PCR Master Mix; Qiagen, Hilden, Germany), 2 µM of each primer and 2 µl of primary PCR product, with a final reaction volume of 10 µl.

Three mixed combinations of *Haemoproteus* sp. hSPMAG12 and *Plasmodium* spp. were prepared to use as positive controls for detection of possible co-infections in penguins: hSPMAG12 × pCXPIP09; hSPMAG12 × pSGS1; and hSPMAG12 × pNYCNYC02. The sample with hSPMAG12 was derived from one of the African penguins (this study, Table 1, sample No. 1b). The three *Plasmodium* spp. samples (pCXPIP09, pSGS1 and pNYCNYC02) were derived from two Magellanic penguins and one Humboldt penguin (*Spheniscus humboldti*), respectively (see Additional file 1: Table S1 for sample information). These blood samples were obtained from two different facilities, one located in central Japan and one located in southwest Japan. In order to minimize any possibility of preferable DNA amplification of one or the other parasite, these positive samples were chosen upon confirmation that the parasitemias were similar, all being less than 1/10,000. *Plasmodium* sp. pCXPIP09 from a Magellanic penguin was also included as a positive control for single infection of parasites belonging to this genus. The three *Plasmodium* spp. lineages, which were used as positive controls, are lineages that have been frequently detected from captive penguins in Japan (our unpublished data), hence are suitable as positive controls for detection of possible co-infections in penguins.

## Results

No false amplifications of the positive control nor cross-amplifications of DNA from other parasites were observed in both negative controls and tested samples during this study. All four penguins and the black-tailed gull were positive for *Haemoproteus* sp. by both PCR and microscopical examination (Table 1). All eight penguin samples were positive using the concentration of 200 ng DNA, but only four samples were positive using 50 ng DNA (Table 1). Both samples from the black-tailed gull were positive using 50 ng DNA.

All detected sequences were identified as *Haemoproteus* sp. hSPMAG12, which is the lineage previously found in a captive Magellanic penguin in Japan (GenBank: AB604310). The same sequence was obtained in two independent tests. This lineage is most similar to *Haemoproteus* sp. hLARCRA02 from a black-tailed gull (2 bp difference or 0.2%) and *Haemoproteus* sp. hNUMPHA01 from a whimbrel (4 bp difference or 0.7%), both first detected from rescued birds [27] at the same facility as the black-tailed gull in this study.

**Fig. 1:**
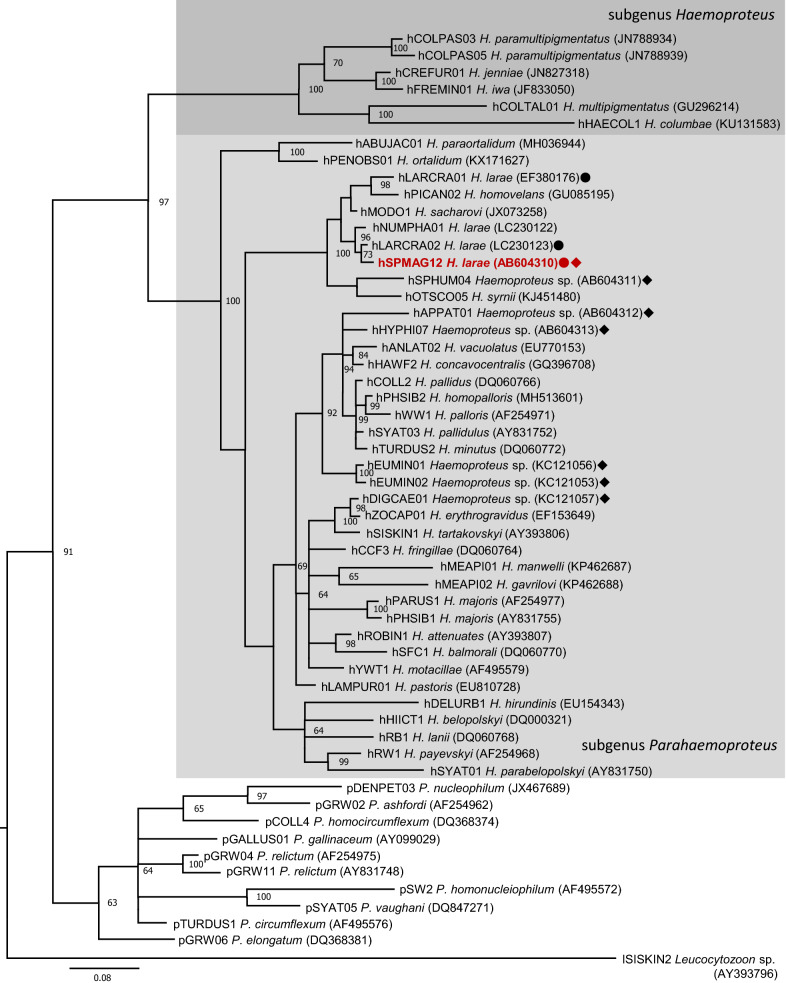
Bayesian phylogenetic analysis of *cytb* gene lineages (470 bp) of avian haemosporidian parasites, rooted with *Leucocytozoon* species lineage lSISKIN2. Posterior clade probabilities of > 0.60 were indicated. The branch lengths are drawn proportionally to the amount of change according to the substitution model applied. *Key*: dark grey area, species of subgenus *Haemoproteus*; light grey area, species of subgenus *Parahaemoproteus*; diamond, *Haemoproteus* spp. lineages derived from penguins in previous studies (see Table 2); circle, *Haemoproteus* spp. lineages derived from *Larus* sp. gulls in previous studies (see Table 2); red letters, lineage derived in this study

When translated into proteins, the lineages hSPMAG12, hLARCRA02 and hNUMPHA01 were identical. Phylogenetic analysis showed that *Haemoproteus* sp. hSPMAG12 was placed in the well-supported clade of parasites belonging to the subgenus *Parahaemoproteus* (Fig. 1).

Multiplex PCR for all penguin samples showed only a single signal for *Haemoproteus* sp. DNA amplification. Two of the three positive control experimental mixes correctly showed two signals for *Plasmodium* sp. and *Haemoproteus* sp. However, only the *Plasmodium* sp. signal was detected for the hSPMAG12 × pSGS1 mixed infection. The single infection positive control for *Plasmodium* sp. pCXPIP09 showed a single signal. The negative control showed no amplification.

**Fig. 2:**
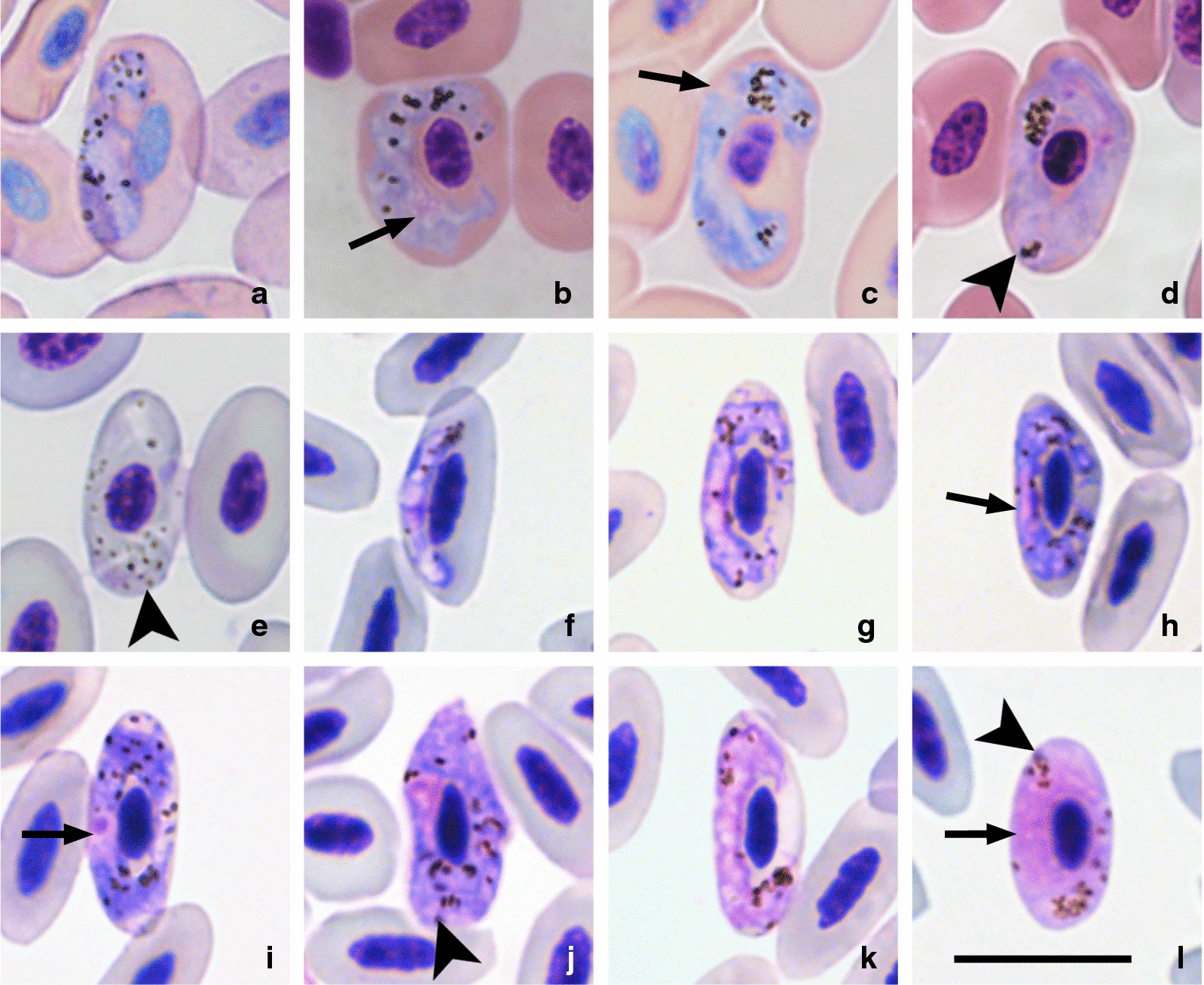
Gametocytes of the lineage hSPMAG12 of *Haemoproteus larae* from the blood of African penguins (*Spheniscus demersus*) (**a**, **c**, **e**; images **a** and **e** are from the same bird individual), Magellanic penguins (*S. magellanicus*) (**b**, **d**) and black-tailed gulls *Larus crassirostris* (**f–l**). Young gametocytes (**a**, **f**), macrogametocytes (**b**, **g**, **h**, **i**, **j**) and microgametocytes (**c**, **d**, **e**, **k**, **l**). *Key*: arrows, gametocyte nuclei; arrowheads, pigment granules. Stained with Hemacolor®. *Scale-bar*:10 µm

One gametocyte was found by microscopical examination in each of the five penguin samples (Table 1, Fig. 2a–e), two of which were from the same individual (Fig. 2a, e). Four of these records were in parallel with PCR positive results using 50 ng DNA. Only one sample (bird No. 2b, Table 1) of an African penguin was positive by microscopical examination, but negative by PCR-based testing using DNA at a concentration of 50 ng. Numerous gametocytes were found in both samples from the black-tailed gull; intensity of parasitemia was 10 and 12 parasites per 1000 erythrocytes in these samples, respectively.

As only one gametocyte was found in each penguin sample (5 gametocytes were visualised in total) during microscopical examination (Fig. 2a–e), parasite species identification by morphology alone was not possible. Meanwhile, blood films from the black-tailed gull contained numerous gametocytes (Fig. 2f–l), which were morphologically identified as *H. larae*, the haemoproteid originally described from a black-headed gull in Kazakhstan [8, 38]. The gametocytes found in the penguin samples were indistinguishable from those of the black-tailed gull, in which the same molecular lineage hSPMAG12 was detected. Merging of these two information sources (i.e. sequence data and gametocyte morphological characters) provided the opportunity to add molecular characterization to the morphological description of *H. larae*, infecting the bird species examined during this study.

### Description of gametocytes *Haemoproteus* (*Parahaemoproteus*) *larae* found in penguins and black-tailed gulls

*Hosts*: The lineage hSPMAG12 was found in the African penguin (*Speniscus demersus*), Magellanic penguin (*S. magellanicus*) and black-tailed gull (*Larus crassirostris*).

*Locality*: Japan.

*Voucher material*: Representative blood films (MPM Coll. No. 21620–21624, 21628, 21629) were deposited in Meguro Parasitological Museum, Tokyo, Japan.

*Representative DNA sequences*: Mitochondrial *cytb* lineage hSPMAG12 (478 bp, GenBank: AB604310).

### Description

[Based on 5 specimens; Fig. 2.] Main diagnostic characters: gametocytes grow around the nuclei of infected erythrocytes and do not displace them laterally (Fig. 2b–l). Growing gametocytes often do not touch the nuclei of erythrocytes (Fig. 2a–d, e–h, i, k). Advanced gametocytes markedly enclose the nuclei with their ends (Fig. 2b, c, g, h) and finally completely encircle the nuclei (Fig. 2d, i, j), occupying all available cytoplasmic space in the erythrocytes (Fig. 2j, l). Growing gametocytes often are irregular in outline (Fig. 2a–c, g, h). Pigment granules are numerous (ranging between 17 and 34), roundish or oval, usually of medium size (< 1µm in length). Details of morphology of the reported macro- and microgametocytes (Fig. 2) agree well with the description of gametocytes from the type vertebrate host, the black-headed gull (*Larus ridibundus*) examined at the type-locality (south Kazakhstan) [8] and are not repeated here.

### Remarks

**Fig. 3:**
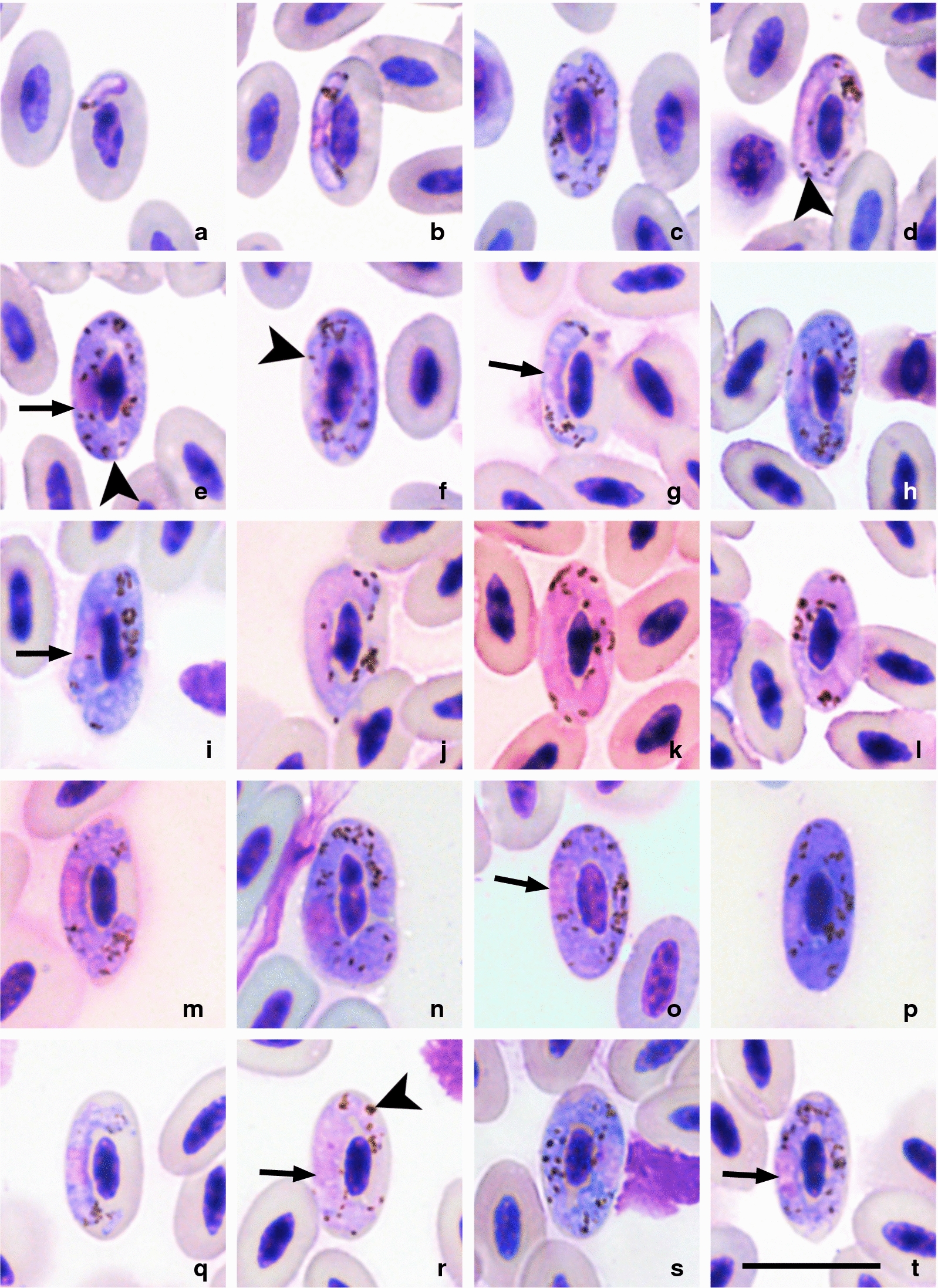
Gametocytes of the lineages hLARCRA01 (**a**–**f**, **m**–**p**), hLARCRA02 (**g**–**l**) and hNUMPHA01(**q**–**t**) of *Haemoproteus larae* from the blood of black-tailed gulls (*Larus crassirostris*) (**a**–**l**), mew gulls (*Larus canus*) (**m**–**p**) and whimbrels﻿ (*Numenius phaeopus*) (**q**–**t**). Young gametocytes (**a**, **b**, **g**, **q**), macrogametocytes (**c**, **e**, **f**, **h**, **i**, **m**–**p**, **s**, **t**) and microgametocytes (**d**, **j**–**l**, **r**). *Key*: arrows, gametocyte nuclei; arrowheads, pigment granules. Stained with Hemacolor®. *Scale-bar*: 10 µm

Yakunin [38] briefly described *H. larae* from the black-headed gull but did not provide illustrations. GV found the same parasite in the type-host in southern Kazakhstan (type-locality) in 1986 and designated voucher material (accessions 1526.86–1527.86, Nature Research Centre, Vilnius, Lithuania), which was used for re-description of *H. larae* (see p. 412 in [8]). Comparison of this material with gametocytes of the lineage hSPMAG12 leaves no doubt that this is the same organism based on morphological characters of its blood stages. Due to markedly circumnuclear gametocytes and numerous pigment granules (see description above and Fig. 2), *H. larae* is readily distinguishable from other haemoproteids of charadriiform birds [8]. It is important to note that gametocytes of the molecularly closest two *cytb* lineages of *Haemoproteus* parasites, hNUMPHA01 (Fig. 3q–t) and hLARCRA02 (Fig. 3g–l), as well as hLARCRA01 (Fig. 3a–f, m–p) detected from *Larus* spp. gulls are morphologically indistinguishable from those of *H. larae* (hSPMAG12) (compare Fig. 2 with Fig. 3) and are considered as different lineages of this species due to their similarity in partial *cytb* gene sequences (Fig. 1).

## Discussion

To the best of our knowledge, this is the first report to document the development of *Haemoproteus* parasite gametocytes in penguins (Sphenisciformes). There have been reports of *Haemoproteus* infection based on PCR diagnostics from blood and tissue samples [18, 19]. However, there was no evidence of the development of the parasites to the gametocyte stage in any of these and other studies. To be transmitted from an avian host to arthropod vectors, haemosporidian parasites must reach the gametocyte stage, which is the only stage infective for vectors. Otherwise the avian host would be a dead end of infection [26, 39]. The key result of this study is that penguins are competent hosts of *Haemoproteus*, which produce gametocytes in penguins. This indicates that vectors can be theoretically infected after feeding on penguins, in other words, from this point of view, these birds can be reservoir hosts of haemoproteosis.

Because of the incredible susceptibility to avian malaria caused by many *Plasmodium* species, numerous studies have tested wild and captive penguins for these pathogens in the past, and there are also some studies that detected *Leucocytozoon* in these birds [5, 6]. Meanwhile, there are only a few publications reporting *Haemoproteus* infections in penguins by PCR-based testing ([18, 19]; Table 2). These studies usually considered the lack of gametocyte detection in the blood of PCR-positive birds as a sign of possible abortive infection [21, 40]. This is possible because not all haemosporidian parasites are able to complete exo-erythrocytic development and reach gametocytes stage in non-competent hosts, resulting in abortive development and dead end of infection [21, 26]. Abortive development might occur mainly in two scenarios. First, vectors might inoculate sporozoites in resistant or partly resistant birds, in which they cannot develop. Haemosporidian sporozoites have been reported in circulation and bird organs [8, 41, 42], and an experimental observation showed that sporozoites can persist for several days and even for roughly a week in such avian hosts after initial infection [41]; thus, they can be templates for PCR amplification, which is a highly sensitive tool and detects even few sporozoites in a blood sample [43]. Another case of abortive infection might occur if the sporozoites successfully reach the internal organs and start exoerythrocytic merogony, which either cannot be completed or the resulting exo-erythrocytic merozoites are unable to penetrate blood cells and develop gametocytes [23, 26, 39]. It is interesting to note that possible exo-erythrocytic meronts of haemoproteids have been detected in three captive penguins in a previous study in Japan [20], but gametocytes were not found. Additionally, tissue stages were reported, but the provided evidence was insufficient to prove that these stages certainly belong to *Haemoproteus* species. In all three infected penguins, acute pathological symptoms were observed, including severe necrosis and inflammation in various organs, ultimately leading to death. These authors assessed the exo-erythrocytic meronts in the liver, which have not often been reported as a site of development of tissue stages in *Haemoproteus* species [6, 8]. However, a recent study detected megalomeronts of *Haemoproteus majoris* in the liver of infected birds [44]. It is worth noting that exo-erythrocytic meronts were also attributed to *Haemoproteus* sp. in Australian little penguins (*Eudyptula novaehollandiae*), in a study which combined histological observations and PCR-based results [21]. However, the same samples were later re-tested and were negative for *Haemoproteus* sp., but instead positive for *Toxoplasma gondii* [45], although the visualised tissue stages appeared similar to cytomeres of *Haemoproteus* [19] rather than cysts and cystozoites of *Toxoplasma* sp. A final conclusion regarding the reasons behind the penguins’ mortality cannot be drawn based on the available information due to possible co-infection with both *Haemoproteu*s and *Toxoplasma* species in Australian little penguins. This case shows that, even when addressed in combination with histological and PCR-based testing, the conclusions about parasite virulence must be done carefully in naturally infected birds. Hence, the report from Ishikawa & Hasegawa [20] should also be addressed with caution.

A possible explanation for the previously rare reports of *Haemoproteus* sp. gametocytes in penguins might be due to the very low parasitemia during chronic infection, as was the case in the present study. In deceased penguins, *Plasmodium* spp. parasitemia is often high [6, 46–48]. However, there are also reports of extremely low parasitemia in some ill individuals [49], and this may result in false-negative results. Only one gametocyte was found in each penguin by checking two blood films per individual. Such low parasitemia is difficult to detect not only during microscopical examination, but also by PCR-based methods. Several previous studies used 50 ng of DNA for PCR [28, 32, 50]. In penguin No. 1 (Table 1), we collected blood four times and obtained a PCR positive signal (using a DNA template of 50 ng) only once when gametocytes were also seen in the blood. Meanwhile, for the other three collected blood samples, PCRs using the same DNA extraction were all negative, but positive using 200 ng of DNA. The commonly applied nested-PCR protocol [28] used in this study might have amplified DNA of newly injected sporozoites (new infections), as mentioned above [43]. This may be a possibility especially if the detected lineage is prevalent among biting midges near the penguin enclosures. There is also a possibility that rather than a new infection, the same parasite persisted in this individual penguin, in which parasitemia may have been fluctuating. With currently available information, it is impossible to distinguish which of the above scenarios is more likely. It is worth noting that blood was collected twice and gametocytes were detected in both samples in penguin No. 2 (Table 1). However, one sample was negative by PCR using 50 ng, but positive using 200 ng DNA template. The parasitemia might be lower than the PCR detection limit using 50 ng DNA template. These findings show that in some host-parasite associations, such as penguins and *Haemoproteus* parasites, PCR-based detection may need a higher DNA concentration in order to detect parasites during very low parasitemia.

The low parasitemia in penguins might be in part due to prophylactic medication against malaria. In many zoos and aquariums, most penguin species are kept outdoors in Japan, with the exception of rare species such as the king penguin that need lower environmental temperatures for maintaining in aquariums [51]. Because penguins are often maintained in an open-air environment where free access of vectors is possible, these birds are often administrated with anti-malarial drugs as prophylactic measures [5, 51]. Medication does not necessarily prevent malaria infection, but does reduce the disease severity [5]. Treatments using antimalarial drugs such as primaquine and atovaquone/proguanil hydrochloride have also been shown to be effective in reducing the parasitemia intensity of *Haemoproteus* spp. [22, 52]. Such effects may also influence haemoproteid parasitemia in penguins in this study, although details regarding the medication protocols were not available for the birds examined. During monitoring of the epidemiology of avian haemosporidiosis in captive penguins, information on infection prophylaxis and its possible influence on parasitemia should be considered.

Co-infections of haemosporidian parasites belonging to the genera *Haemoproteus* and *Plasmodium* are common in wildlife and even predominate in some bird populations [53–55]. It is well established that although PCR is a very sensitive and reliable diagnostic tool, it can be insufficiently sensitive to report co-infections of haemosporidians, and some such infections might be overlooked [36, 55–57]. The vast majority of reported haemosporidian infections in penguins are *Plasmodium* parasites, and there have only been a few reports of *Haemoproteus* spp. [5, 6]. Depending on the parasite species, it is sometimes difficult to morphologically distinguish between *Plasmodium* spp. and *Haemoproteus* spp. gametocytes, especially if only young (growing) gametocytes are present in the circulation [8]. In the present study, a combination of two multiplex PCR protocols were tested in order to confirm that the observed parasites were *Haemoproteus* spp., rather than *Plasmodium* spp. which are more common in penguins. No DNA amplification for *Plasmodium* spp. was confirmed, and all samples showed a signal for *Haemoproteus* sp. at 533 bp, confirming that all tested penguins had single infections. However, although these multiplex PCR protocols were more sensitive in detection of co-infections [36, 37], it is important to note that some combinations of lineages might be more difficult to distinguish than others [56]. Hence, PCR results need to be carefully analyzed especially when linking these results to morphological data. In one of our positive experimental controls (the combination hSPMAG12 × pSGS1), we found only the *Plasmodium* sp. signal at 378 bp. This might be due to an imbalance in the parasitemia of the two parasites. All positive controls were chosen upon confirmation that parasitemia was less than 1/10,000. However, besides the fact that both samples used for preparation of the mixture have extremely low parasitemia, we were unable to confirm the exact parasitemia in each sample used. Another possibility is that primers may favour and bind with DNA of parasites belonging to one genus over another [37]. These might result in an imbalanced reaction where DNA of one parasite may be more favourable for amplification [55].

In this study, we detected only the lineage hSPMAG12 in all of the penguins as well as the black-tailed gull. All samples were tested twice, each by a different person in order to ensure that there was no cross-contamination among other samples in the laboratory. Penguin samples and black-tailed gull samples were never handled or processed together. The same results were obtained in both trials, and no amplification was observed for the negative control bands, confirming that contamination did not occur. This lineage has only previously been detected from one Magellanic penguin kept in an aquarium located in the north part of Japan in 2006 (GenBank: AB604310; location of the individual has been confirmed by YS *via* personal communication). The penguins positive for *Haemoproteus* sp. in this study were all hatched and raised in Japan (Table 1), meaning that the infections occurred locally in Japan.

Because only one gametocyte was found per blood film in penguins, it would be hardly possible to identify parasite species based solely on this information. However, all reported gametocytes in penguins belonged to the same lineage (hSPMAG12) as in the black-tailed gull in this study. In addition, gametocytes of the penguins (Fig. 2a–e) were morphologically indistinguishable from gametocytes of the black-tailed gull (Fig. 2f–l) which has been identified as *H. larae*. Morphological features of *H. larae* gametocytes in this study (Fig. 2) coincided with former descriptions of this species, including gametocytes seen in the type vertebrate host (black-headed gull) [8, 38, 58]. The combination of molecular and morphological identification strongly suggests that the penguins are infected with the same parasite species. It is important to note that morphological variations of blood stages are known to exist when the same *Plasmodium* parasite occurs in birds belonging to different orders [8, 59], but this issue remains uninvestigated in *Haemoproteus* parasites. In this study, mature gametocytes of *H. larae* seem to retain the main diagnostic characters when they develop in penguins. However, additional research is needed to address this issue in detail due to the small number of gametocytes recorded in penguins. Meanwhile, closest *Haemoproteus* lineages hNUMPHA01 and hLARCRA02 as well as the lineage hLARCRA01 have gametocytes which are morphologically indistinguishable from *H. larae* and were identified as members of this parasite morphological group (Fig. 3). Similar intraspecific variations have been reported in the relatively well-studied morphospecies *Haemoproteus majoris* [59], *Plasmodium relictum* [60] and other avian haemosporidians, in which morphologically defined species are known to consist of multiple closely related *cytb* lineages. While there have been a few reports of *H. larae* in gulls, these reports are all solely based on morphological identification [61], and future investigation using a combination of molecular and morphological methods may reveal other molecular lineages of *H. larae*.

It is important to note that the phylogenetic analysis placed the lineage hSPMAG12 of *H. larae* well within the subgenus *Parahaemoproteus* clade (Fig. 1). Recent experimental studies have proved that *Culicoides* biting midges transmit all tested *Parahaemoproteus* species [62, 63], suggesting that biting midges are likely vectors of *H. larae* as well. These biting midges may carry the parasite from wild gulls to captive penguins or *vice versa*, although investigation on the presence of biting midges at the aquariums remain insufficient.

Penguin No. 1 and No. 2 (Table 1) were sampled several times, with a span of 15 and seven months between the first and last sampling date, respectively. As mentioned above, the same lineage hSPMAG12 was detected from all of these samples using 200 ng of DNA template. All *Haemoproteus*-infected penguins did not show external signs of illness during the period of blood collection. This may possibly indicate that the parasite was able to persist within the host for over a year without exhibiting any apparent symptoms. However, the possibility that a new infection occurred cannot be ruled out, and the available information remains insufficient to determine which scenario is more plausible.

If parasites did indeed persist within the penguins, such asymptomatic long-lasting infections indicate that penguins might act as reservoirs of *H. larae* (hSPMAG12) infection. Because the intensity of parasitemia was extremely low in these penguins, a question remains regarding whether transmission from these individuals to vectors is possible. A previous study using human malaria *Plasmodium falciparum* confirmed the development of oocysts in mosquitoes that fed on host blood with submicroscopic gametocytemia [64]. Other studies using *P. relictum* reveal that parasitemia and transmission may fluctuate in response to various factors such as the exposure to mosquitoes and time of day [65, 66], suggesting that parasitemia alone may not be a good predictor of transmission [65]. It remains unknown whether similar patterns are possible in *Haemoproteus* parasites. Further research is required to determine whether penguins are capable of being reservoirs of haemoproteosis and might be a source of *H. larae* (hSPMAG12) for transmission to other birds *via* vectors.

Meanwhile, the intensity of parasitemia was 1.0–1.2% in the black-tailed gull in this study, and such parasitemia is high enough for biting midges to develop sporozoites [63]. Like other gull species [8], the black-tailed gull is a natural host of *H. larae*. This species, and possibly other gull species in Japan, may act as a reservoir of the parasites. It is noteworthy that this species often visits outdoor aquariums and could be a source of infection to vectors. Interestingly, the type host of *H. larae*, the black-headed gull, is common in Japan and also frequently visits such aquariums. Although not as frequent, shorebirds such as whimbrel, in which *H. larae* (hNUMPHA01) was detected, may also visit such open-air aquariums. Penguins, especially *Spheniscus* spp., are often kept outdoors in Japan, as was the case in two aquariums in this study. There are no obstacles for vectors to feed on both free-ranging gulls and captive penguins at these aquariums. *Haemoproteus larae* (hSPMAG12) could therefore be shared between the free-ranging gulls and captive penguins, with negligible genetic variation in their partial *cytb* gene sequences (i.e. hLARCRA01, hLARCRA02 and hNUMPHA01) that may have occurred in the process of different host shifts.

Further investigation is required to better understand the transmission and life-cycle of haemoproteids in penguins. Examination of vectors and wild birds, particularly charadriiform birds such as gulls, near penguin aquariums is needed to evaluate the rate of possible transmission from wild birds and pathogenicity of the infection. Light parasitemia might indicate adaptation between the host and the parasite resulting in a low virulence, but it might also be a result of antimalarial medication used.

If competent vectors are available, *H. larae* could potentially spread between penguins even without access to wild birds. Many preventative measures against avian malaria have been developed by using mosquito-free indoor facilities and by setting wind curtains and mosquito traps [5]. However, these measures have little effect or have not been tested against biting midges, the likely vectors of *H. larae* (hSPMAG12). In order to prevent the spread of haemoproteosis, the control of biting midges is also necessary.

## Conclusions

Penguins are susceptible to haemoproteosis and are competent hosts of *Haemoproteus* species, which complete their life-cycle and produce gametocytes in these birds. In other words, not only wild birds, but also penguins might be a source of *Haemoproteus* infection in penguin aquariums. In Japan, the lineage hSPMAG12 of *H. larae* parasitize penguins. Wild black-tailed gull (and probably other gull species) are natural reservoir hosts of this infection. Molecular characterization of *H. larae* parasitizing the black-tailed gull and penguins was developed and can be used for detection of this infection. Phylogenetic analysis suggests that *Culicoides* biting midges should be incriminated in haemoproteosis transmission in penguins. Virulence of *Haemoproteus* parasites in penguins needs additional investigation. While *Haemoproteus* infection may be non-lethal in penguins, particularly during antimalarial medication, these birds can be a source of infection for other captive penguins, including young or weak individuals which may show clinical symptoms. As many species of penguins are endangered and protected, numerous facilities in Japan keep penguins for conservation purposes. This study shows that *Haemoproteus* species should be considered as penguin pathogens, which need attention in order to improve *ex-situ* conservation of this unique bird group. We call for further investigations targeting the biting midges as likely haemoproteosis vectors, as well as the wild birds as possible reservoir hosts of *Haemoproteus* infections in penguins.

## Supplementary information

**Additional file 1: Table S1.** Information on *Plasmodium* spp. samples used to prepare mixed-infections.

## Data Availability

The data supporting the conclusions of this article are included within the article and its additional file. Representative preparations of blood stages from penguins (MPM Coll. No. 21620–21624), mew gull (MPM Coll. No. 21625), black-tailed gull (MPM Coll. No. 21626–21629) and whimbrel (MPM Coll. No. 21630) were deposited in the Meguro Parasitological Museum, Tokyo, Japan.

## Abbreviations

DNA: deoxyribonucleic acid
EDTA: ethylene-diamine-tetra-acetic acid
PCR: polymerase chain reaction
*cytb*: cytochrome *b*
