# Supplementary material for: Penguins are competent hosts of Haemoproteus parasites: the first detection of gametocytes, with molecular characterization of Haemoproteus larae
Source: Parasit Vectors. 2020 Jun 12;13:307. doi: 10.1186/s13071-020-04176-1 (PMC7291633; doi:10.1186/s13071-020-04176-1)
Supplement: Supplementary file 1 — Additional file 1: Table S1. Information on Plasmodium spp. samples used to prepare mixed-infections. [file 13071_2020_4176_MOESM1_ESM.docx]

**Additional file 1: Table S1**. Information on *Plasmodium* spp. samples used to prepare mixed-infections for PCR-based testing.

| Lineage | Bird species | Facility | Date of hatching | Sampling date | | Intensity of  parasitemia |
| --- | --- | --- | --- | --- | --- | --- |
| pCXPIP09 | *Spheniscus magellanicus* | Aquarium C | 1997-05-20 |  | 2017-10-19 | <1/10,000 |
| pSGS1 | *S. magellanicus* | Aquarium C | 2003-05-24 |  | 2017-10-19 | <1/10,000 |
| pNYCNYC02 | *S. humboldti* | Aquarium D | 2002-04-29 |  | 2016-02-04 | <1/10,000 |
